# BmToll9-1 Is a Positive Regulator of the Immune Response in the Silkworm *Bombyx mori*

**DOI:** 10.3390/insects15090643

**Published:** 2024-08-27

**Authors:** Jisheng Liu, Weijian Chen, Jinrong Situ, Jiaxuan Li, Jiahua Chen, Minchun Lai, Fengyi Huang, Baoqi Li

**Affiliations:** School of Life Sciences, Guangzhou University, Guangzhou 510006, China

**Keywords:** *Bombyx mori*, Toll receptor, BmToll9-1, RNA interference, antimicrobial peptides, immune response, *Escherichia coli*

## Abstract

**Simple Summary:**

Insects rely on innate immunity to defend against invading pathogens in nature. BmToll9-1 is a receptor in the immune pathway of silkworms. BmToll9-1 is abundantly present in the midgut tissue of silkworm larvae. Silencing of BmToll9-1 results in smaller larval and cocoon bodies. Concomitantly, the expression of most downstream genes in the immune pathways is reduced. The antibacterial activity of hemolymph is also inhibited. The findings reveal that BmToll9-1 plays a positive role in regulating the development and immune response in silkworm.

**Abstract:**

Toll receptors are involved in the development and innate immunity of insects. BmToll9-1 is an important immune receptor in the Toll pathway. Previous studies have focused on its role as a receptor in immune response. In this study, we aimed to investigate the role of BmToll9-1 as a regulator in the immune response. The expression profiles demonstrated that *BmToll9-1* was predominantly expressed in the midgut. RNA interference (RNAi) of *BmToll9-1* was found to be effective in the midgut via the injection of dsRNA, which resulted in smaller and lighter larvae and cocoons. Most signaling genes in the Toll pathway and downstream effector genes were downregulated after the RNAi of *BmToll9-1*. The hemolymph from *BmToll9-1*-silenced larvae showed decreased antibacterial activity against *Escherichia coli*, either in growth curve or inhibition zone experiments. The above results indicate that BmToll9-1 might be positively involved in the immune pathway of silkworm. As a positive regulator, BmToll9-1 might function mainly in the gut to maintain microbial homeostasis to regulate the growth of silkworms. Silencing of *BmToll9-1* downregulates the signaling genes in the Toll pathway and antimicrobial peptide (AMP) production, resulting in decreased antibacterial activity in the hemolymph.

## 1. Introduction

The silkworm *Bombyx mori*, an economically important insect with a long history in China, not only provides silk for textiles but also has significant scientific research value [1]. Insects resist the invasion of exogenous pathogens primarily through their innate immune system [2], which synthesizes and secretes antimicrobial peptides (AMPs), mediated by the Toll and immune deficiency (IMD) signaling pathways [3]. The Toll and IMD signaling pathway are triggered by Gram-positive bacteria and fungi as well as Gram-negative bacteria, respectively [4].

The first Toll receptor was discovered during studies of embryonic development in *Drosophila melanogaster* [5]. Insect Toll is a type I transmembrane protein that can be divided into three parts: extracellular, transmembrane, and intracellular regions. The extracellular region is rich in leucine-rich repeats (LRRs), which play a role in ligand recognition. The intracellular region contains a conserved Toll/interleukin-1 receptor (TIR) domain, which interacts with downstream molecules such as myeloid differentiation factor 88 (MyD88) [6,7]. MyD88 contains a death domain that forms a complex with Tube and Pelle when Späetzle binds to the extracellular domain of the Toll receptor. This leads to the phosphorylation of Cactus, which interacts with Rel proteins, Dorsal and Dif. Upon phosphorylation, Cactus dissociates from Dorsal and Dif, allowing Rel proteins to translocate from the cytoplasm into the nucleus. As a result, Rel proteins activate AMP genes [8].

A subsequent study found that the number of Toll receptors in insects varies depending on species, receptor subtype, developmental stage, and environmental conditions [3]. For example, there are 9 Toll receptors in *D. melanogaster* [9,10], 5 Toll receptors in *Apis mellifera* [11], and 11 Toll receptors in *Anopheles gambiae* [12]. *B. mori*, a model organism of Lepidoptera, was found to have 14 Toll-related receptors [8]. The expression profiles of Toll genes in *B. mori* show that *BmToll3-3*, *BmToll7-2*, and *BmToll7-3* are enriched in the ovary and midgut. *BmToll9-1* has abundant expression in the midgut, while *BmToll9-2* has enriched expression in Malpighian tubules and brain [13].

The current research indicates that Toll receptor genes in silkworms are induced by *Escherichia coli*, *Staphylococcus aureus*, and *Beauveria bassiana* [14,15]. The expressions of Toll receptor genes in silkworms were altered upon invasion by exogenous pathogens [14]. *BmToll3-2*, *BmToll3-3*, *BmToll10-2*, and *BmToll10-3* were significantly upregulated by fungi and *E. coli* induction [1]. We previously found that the expression of *BmToll9-1* in the midgut was significantly suppressed after the injection of double-stranded RNA (dsRNA) and lipopolysaccharide (LPS) into silkworm larvae [13]. The expression level of *BmToll9-1* in the midgut significantly increased when silkworm was infected with *E. coli* and *B. bassiana* [14]. We also found that in the silkworm-derived Bm5 cells overexpressing *BmToll9-1*, RNA interference (RNAi) machinery gene *Dicer-2* was upregulated; immune genes in the IMD and Janus kinase/signal transducers and activators of transcription (Jak/Stat) pathway, as well as AMPs genes, were repressed by LPS [16]. In our recent study, oral infection of *E. coli* and its main cell wall component LPS, as well as *S. aureus* and its main cell wall component peptidoglycan (PGN), significantly induced the expression of *BmToll9-2* [17]. The above results suggest that different Toll receptor genes play different roles in the immune response. Notably, phylogenetic tree analysis revealed that *BmToll9-1* and *BmToll9-2* are closely related to mammalian *Toll-like receptor 3* (*TLR3*) and *TLR4* [18], suggesting that these two receptors are associated with the immune response.

Here, we focused on elucidating the role of the BmToll9-1 receptor in regulating the development and signaling genes in the Toll pathway and downstream AMPs and other effector genes via dsRNA-mediated gene silencing.

## 2. Materials and Methods

### 2.1. Insect Rearing and Tissue Dissection

The “P50” strain of silkworms used in this study was provided by Guangdong Academy of Agricultural Sciences, Guangzhou, China. After hatching, the larvae were reared on fresh mulberry leaves at 25 ± 1 °C, 75 ± 5% relative humidity, and a photoperiod of 12 L:12 D. The silkworm larvae at the 5th instar were selected for dissection and sampling, including epidermis, fat body, midgut, silk glands, Malpighian tubules, and hemocytes. All samples were immediately transferred to RNase-free tubes and stored at −80 °C.

### 2.2. RNA Extraction, cDNA Synthesis, and qPCR

Total RNA was extracted from frozen tissue samples using an RNAiso Plus kit (TakaRa, Kusatsu, Japan) according to the instructions. Genomic DNA was removed via DNase digestion, and first-strand complementary DNA (cDNA) was synthesized using a PrimeScript RT Reagent Kit (Perfect Real Time) (TaKaRa).

GoTaq qPCR Master Mix (Promega, Madison, WI, USA) was used for qPCR amplification, using the midgut cDNA of the 5th-instar larvae as a template. The thermal reaction was performed in a CFX Connect Real-Time System (Bio-Rad, Hercules, CA, USA) as follows: 95 °C for 2 min, 40 cycles of 95 °C for 15 s, and 57 °C for 30 s. A dissociation step from 65 °C to 95 °C was added after the thermal cycles. Primers for detecting *BmToll9-1*, signaling genes, and effector genes are listed in Table 1. Translation initiation factor 4A (BmTIF4A) and translation initiation factor 3 subunit 4 (BmTIF3s4) were used as the reference genes [19]. The normalization of expression levels was performed after geometric averaging of the reference genes. The relative expression levels of target genes were determined with the 2^(−ΔΔCT)^ method [20]. All qPCR reactions were performed using three biological replications. For each biological replication, three technical replications were carried out.

### 2.3. RNAi Protocol

RNAi was performed as described in our previous report [21]. To produce the template DNA, the T7 RNA polymerase promoter sequence (Table 1) was added to the primers before PCR amplification. The purified PCR fragment was used for dsRNA synthesis with T7 RiboMAX Express RNAi (Promega, Madison, WI, USA). dsRNA for green fluorescent protein (dsGFP) was used as a negative control. dsRNA was diluted to 500 ng/μL, and 10 μL of diluted dsRNA was injected on Day 1 into the 5th-instar larvae. Dissected samples were collected at 6, 12, and 24 h after injection.

### 2.4. Antibacterial Activity Assays

To investigate whether RNAi of *BmToll9-1* affected antibacterial activity, the larval hemolymph was isolated at 24 h after RNAi treatment. The hemolymph was boiled at 100 °C for 5 min, then centrifuged at 4 °C at 10,000 rpm for 10 min, and the supernatant was collected and stored at −20 °C [22]. 

For the bacterial growth curve experiment, *E. coli* and *S. aureus* were cultured in Luria–Bertani (LB) medium at 37 °C under shaking at 200 rpm until OD_600_ = 0.3. Then, 80 μL of bacterial culture was mixed with 20 μL of heat-treated hemolymph to a final volume of 100 μL in a 96-well plate. The plate was incubated at 37 °C, and the OD_600_ was measured every hour with an Infinite M Plex microplate reader (Tecan, Männedorf, Switzerland).

For the inhibition zone experiment, the filter paper diffusion method (Kirby–Bauer method) was used to measure the inhibition zone [22]. LB agar medium was melted by heating and then cooled to approximately 50 °C. *E. coli* and *S. aureus* (OD_600_ = 0.3) were added to the medium at a ratio of 1:100 and 1.5:100, respectively, mixed well, and poured into petri dishes. Filter paper discs were placed on the medium, and 20 μL of heat-treated hemolymph was added dropwise. Next, 20 μL of sterile water or Amp (20 ng/μL) was added separately as a negative or positive control. The petri dishes were incubated overnight in a constant-temperature incubator at 37 °C. The diameter of the inhibition zone was observed the next day.

### 2.5. Data Analysis

Statistical analysis was performed using two-way ANOVA combined with Student’s *t*-test using SPSS version 26.0. Data were plotted in GraphPad Prism v10.0 and are expressed as mean ± standard deviation of the replicates of three biological replications. Knockdown or inhibition rate were compared using mean values to the control groups and converted to percentage. 

## 3. Results

### 3.1. The BmToll9-1 Gene Was Effectively Silenced in the Midgut

**Figure 1:**
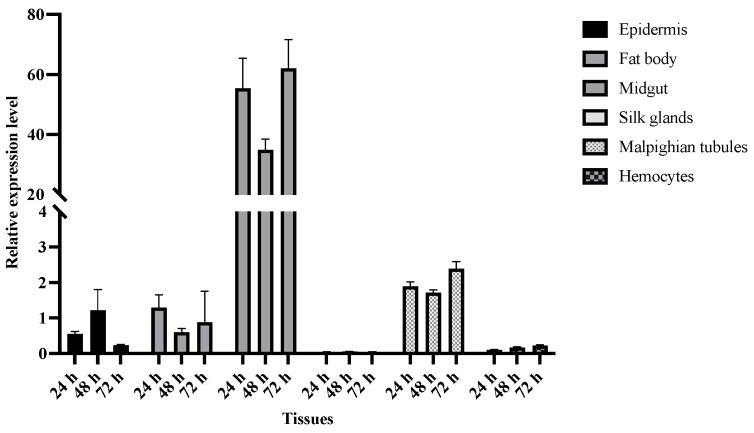
The relative expression of *BmToll9-1* in different tissues of 5th-instar silkworm larvae. The epidermis, fat body, midgut, silk glands, Malpighian tubules, and hemocytes were collected at 24, 48, and 72 h. The relative expression was calculated in all the tissues. Data are represented as means ± standard deviation of three biological replications. For each biological replication, there were three technical replications.

The expression profile indicated that the *BmToll9-1* gene was predominantly expressed in the midgut of the fifth-instar silkworm larvae (Figure 1). On the contrary, the relative expression of the *BmToll9-1* gene in the epidermis, fat body, and Malpighian tubules was low. In the silk glands and hemocytes, the expression was extremely low. Therefore, the midgut was analyzed as the tissue in the subsequent RNAi experiment.

**Figure 2:**
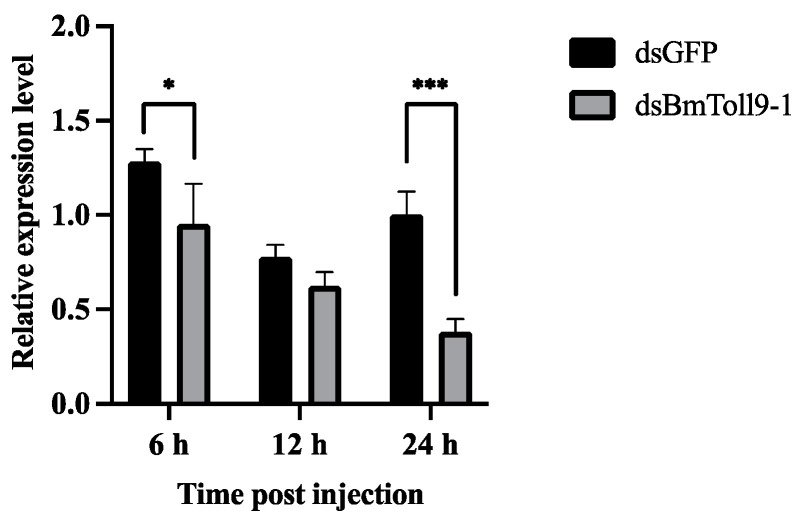
RNAi of *BmToll9-1* gene via dsRNA injection. dsBmToll9-1 was injected to the 5th-instar larvae, and dsGFP served as the control. The relative expression of *BmToll9-1* was detected at 6, 12, and 24 h after dsRNA injection. The relative expression was calculated compared with that of the control. Data are represented as means ± standard deviation of three biological replications. For each biological replication, there were three technical replications. Asterisks indicate significant differences in dsGFP injection groups: * *p* < 0.05, *** *p* < 0.001.

To detect the optimal time for RNAi, the relative expression levels in the midgut were examined at 6, 12, and 24 h after dsRNA injection. It was observed that the significantly highest silencing effect was achieved at 24 h after injection of dsBmToll9-1, where the relative expression of the *BmToll9-1* gene was 64% lower than in the control (injection of dsGFP) (Figure 2).

### 3.2. RNAi of BmToll9-1 Gene Affected the Growth of the Silkworm Larvae and Cocoons

**Figure 3:**
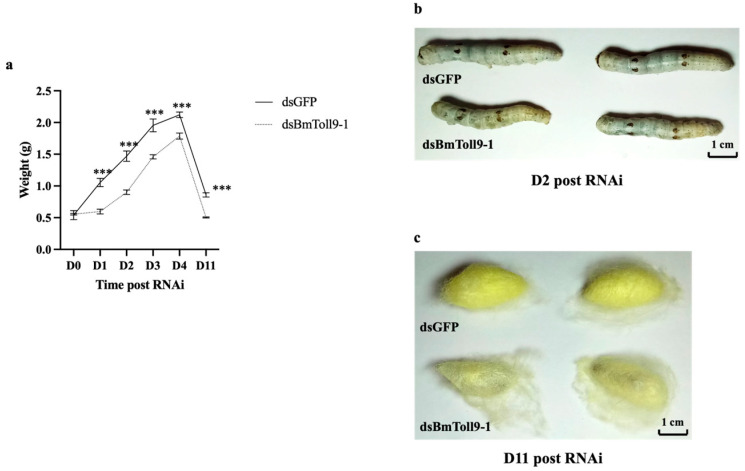
Phenotype observation and weight determination after RNAi of *BmToll9-1*. (**a**) The average weight of silkworm larvae on different days after dsRNA injection. (**b**) The appearance of silkworm larvae at D2 after injection of dsRNA. (**c**) The appearance of silkworm cocoons at D11 after injection of dsRNA. Asterisks indicate significant differences compared with dsGFP injection groups: *** *p* < 0.001.

After the *BmToll9-1* gene was silenced, the larval weight was lighter than in the control (injection with dsGFP). Significant differences in weight were found on Day 1 after injection of dsBmToll9-1, and the biggest differences occurred on Day 3 (Figure 3a). By observing the phenotype, it was found that the *BmToll9-1*-silenced larvae appeared shorter and smaller (Figure 3b). Similarly, the *BmToll9-1*-silenced cocoons were smaller in size and thinner in the silk (Figure 3c). 

### 3.3. RNAi of the BmToll9-1 Gene Reduced the Expression of Signaling GENES in the Toll Pathway

**Figure 4:**
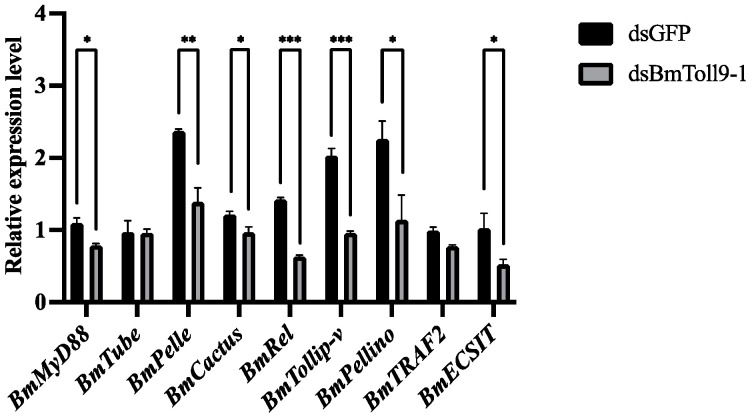
The relative expression of the signaling genes in the Toll pathway after RNAi of *BmToll9-1*. Larvae of the 5th instar were injected with dsBmToll9-1, and dsGFP served as the control. The relative expression was calculated compared with the control. Data are represented as means ± standard deviation of three biological replications. For each biological replication, there were three technical replications. Asterisks indicate significant differences compared with dsGFP injection groups: * *p* < 0.05, ** *p* < 0.01, *** *p* < 0.001.

Considering that the midgut is a barrier tissue and silencing of *BmToll9-1* was achieved in the midgut, midgut samples were collected to detect the expression of the signaling genes in the Toll pathway (Figure 4). Compared with the control group (injection with dsGFP), the signaling genes in the Toll pathway, except *BmTube* and *BmTRAF2*, were significantly downregulated in the RNAi group (injection with dsBmToll9-1). *BmMyD88*, *BmPelle*, *BmCactus*, and *BmRel* were inhibited by 20%, 40%, 21%, and 23%, respectively. *BmTollip-v*, *BmPellino*, and *BmECSIT* were suppressed by 53%, 50%, and 50%, respectively.

### 3.4. RNAi of the BmToll9-1 Gene Reduced the Expressions of the Downstream Effector Genes

**Figure 5:**
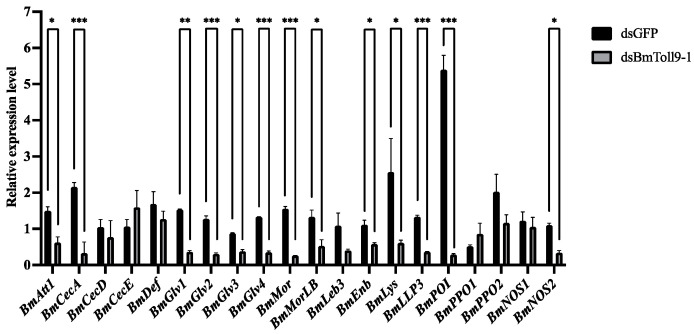
The relative expression of the effector genes after RNAi of *BmToll9-1*. Larvae of the 5th instar were injected with dsBmToll9-1, and dsGFP served as the control. The relative expression was calculated compared with that of the control. Data are presented as means ± standard deviation of three biological replications. For each biological replication, there were three technical replications. Asterisks indicate significant differences compared with dsGFP injection groups: * *p* < 0.05, ** *p* < 0.01, *** *p* < 0.001.

Similarly, midgut samples were used to detect the expressions of 20 effector genes (Figure 5). Compared with those of the control group, most AMP genes were downregulated in the RNAi group. The *Attacin* gene *BmAtt1* was significantly downregulated by 58%. The three *Cecropin* genes were downregulated, but only *BmCecA* was significantly downregulated by 85%. The four *Gloverin* gene expressions were significantly decreased when *BmToll9-1* was silenced, with *BmGlv1* showing the highest reduction of 76%. Both *Moricin* and *Moricin*-like genes *BmMor* and *BmMorLB* showed significant 83% and 60% reductions, respectively. The *Enbocin* gene *BmEnb* was significantly reduced by 48%. Both *Defensin* and *Lebocin* genes *BmDef* and *BmLeb3* were downregulated but not significantly so. 

For the other effector genes, lysozyme *BmLys* and lysozyme-like protein *BmLLP3* genes were significantly reduced by 76% and 72%, respectively. Prophenoloxidase *BmPPO2* and phenoloxidase inhibitor *BmPOI* genes were downregulated, but only *BmPOI* was significantly downregulated by 95%. Nitric oxide synthase *BmNOS2* was significantly reduced by 69%, while *BmNOS1* was downregulated without significant differences compared with the control. 

### 3.5. RNAi of BmToll9-1 Decreased Antibacterial Activity

**Figure 6:**
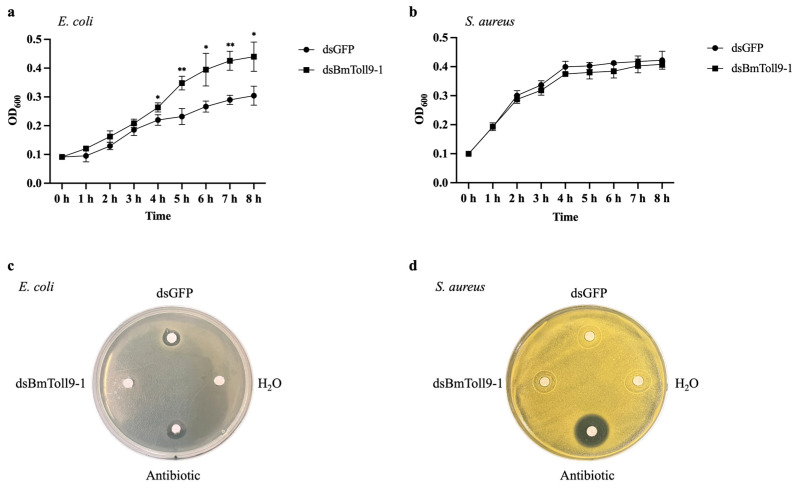
Antibacterial activity assays of *B. mori* hemolymph against *E. coli* and *S. aureus* after RNAi of *BmToll9-1*. Hemolymph was collected at 24 h after dsRNA injection. (**a**,**c**) Bacterial growth curve experiment. (**b**,**d**) Inhibition zone experiment. dsBmToll9-1: hemolymph from larvae injected with dsBmToll9-1; dsGFP: hemolymph from larvae injected with dsGFP; H_2_O: sterile water; antibiotic: ampicillin. Asterisks indicate significant differences compared with dsGFP injection groups: * *p* < 0.05, ** *p* < 0.01.

To further investigate whether silencing of *BmToll9-1* affects the immune response in silkworm larvae, the hemolymph was collected for antibacterial activity assays against the Gram-negative bacterium *E. coli* and the Gram-positive bacterium *S. aureus*. The *E. coli* bacterial growth curve experiment showed that the hemolymph from the *BmToll9-1*-silenced larvae grew better than the control group, with significant differences between 4 and 8 h (Figure 6a). In the inhibition zone experiment, both ampicillin and dsGFP-injected hemolymph showed antibacterial activity in the *E. coli* plate, while sterile water and dsBmToll9-1-injected hemolymph displayed no inhibitory effect (Figure 6b). As for *S. aureus*, the dsBmToll9-1-injected hemolymph showed no significant differences in either bacterial growth (Figure 6c) or inhibition zone (Figure 6d) compared with the dsGFP-injected hemolymph. The above results showed that the hemolymph of the silkworm larvae lost antibacterial activity when *BmToll9-1* was silenced, indicating it might act against Gram-negative bacteria.

## 4. Discussion

It is well known that the Toll signaling pathway plays an important role in the insect immune response [3]. The Toll receptor, an important component of the signaling pathway, transmits signals detected by its extracellular domain to the intracellular compartment, thereby activating the innate immune response of insects. Previous reports have indicated that Toll receptors are involved in the development and innate immunity of insects [6,7]. 

Previous studies on BmToll9-1 have focused its role as a receptor in the immune response. For example, BmToll9-1 responded differently to different microbial infections [14]. We found that LPS inhibited the expression of BmToll9-1 in larvae [13]. BmToll9-1 could bind to BmSpz2 [23]. BmToll9-1 was later proven to be a pattern recognition receptor (PRR) for LPS that shares conserved features with the mammalian TLR4-MD-2-LPS pathway [18]. 

In this study, the role of the BmToll9-1 receptor in regulating development and the Toll signaling pathway of the silkworm was investigated for the first time. Even though it was reported that RNAi efficiency is refractory in silkworms and other lepidopteran insects [24], we successfully characterized the *BmToll9-1* receptor gene using a dsRNA-mediated RNAi technique.

### 4.1. BmToll9-1 May Modulate Gut Homeostasis

*BmToll9-1* being predominantly expressed in the midgut and weakly expressed in other tissues in the larvae (Figure 1) or pupae [13] suggests that BmToll9-1 mainly functions in the gut of silkworm. The successful RNAi of *BmToll9-1* gene in the midgut (Figure 2) resulted in slower growth of the silkworm larvae and pupae (Figure 3). The midgut is the location where digestion occurs after feeding. Thus, the gut epithelium has to cope with an ever-changing intestinal environment to regulate the complex microbial community to achieve immunity and intracellular homeostasis. Silencing of the *BmToll9-1* gene might imbalance the intestinal microbial homeostasis, thus affecting food digestion and intake. Studies on the effect of the gut microbiota on development have been conducted in other insects, such as *D. melanogaster* [25] and *Bombus terrestris* [26]. The regulatory mechanisms of microbial homeostasis in the insect gut have been identified in several insects, which are related to the key genes in the immune pathways [27]. Our successful silencing of *BmToll9-2* inhibited the growth of silkworm larvae [17]. Similar results were observed in our recent study on another receptor in the immune pathways. The RNAi of *BmPGRP-L4* gene also slowed the growth of silkworm larvae [21]. Therefore, it is likely that BmToll9-1 is involved in the development of these larvae by regulating gut homeostasis. 

### 4.2. BmToll9-1 Is Positively Involved in the Toll Pathway

In the Toll pathway, Toll, MyD88, Tube, Pelle, Cactus, and Rel are involved in signal transduction in the cytoplasm [9]. Intercellular components such as Tollip, Pellino, TNF receptor-associated factor-2 (TRAF2), and ECSIT appear to be involved in this process [8]. The successful RNAi of the *BmToll9-1* gene resulted in significant reductions in the above signaling genes in the Toll pathway, except Tube and TRAF2 (Figure 4). These results suggest that BmToll9-1 is positively related to the downstream signaling genes in the Toll pathway. 

The regulation of the *Toll* genes in the signaling pathways has been extensively studied in insects, such as *D. melanogaster* [28] and *Aedes aegypti* [29]. We found that in the silkworm-derived Bm5 cells overexpressing the *BmToll9-1*, the signaling genes in the IMD and Jak/Stat pathways were repressed by LPS [16]. In the Chinese oak silkworm *Antheraea pernyi*, the Toll pathway genes are positively associated. The *Toll* gene was induced by fungus *Nosema pernyi* and Gram-positive bacterium *Enterococcus pernyi*. Additionally, the *MyD88*, *Cactus*, and *Rel* genes were induced by the two above pathogens [30]. The *Toll* gene was induced when honey bees were infected by pathogens, and the expression of its adapter protein *MyD88* was upregulated [11,31]. Infection of *Laodelphax striatellus* with rice stripe virus directly activated the Toll pathway, and the expressions of *Tube*, *MyD88*, and *Dorsal* genes were upregulated in viruliferous planthoppers [32].

### 4.3. BmToll9-1 Positively Regulates the Immune Effectors

In this study, when *BmToll9-1* gene was silenced, most of the AMPs and other immune effectors were down-regulated (Figure 5). At the same time, hemolymph from *BmToll9-1*-silenced larvae showed decreased antibacterial activity against *E. coli*, either in growth curve or inhibition zone experiments (Figure 6). These results suggest that BmToll9-1 is positively related to the production of AMPs. 

Because LPS is the main cell wall component of *E. coli* and BmToll9-1 is the PRR for LPS [18], it is reasonable that the decreased antibacterial activity was effective against *E. coli* but not *S. aureus*. The involvement of AMPs regulation was reported in the silkworms extensively. For example, we found that in the silkworm-derived Bm5 cells overexpressing *BmToll9-1*, AMPs genes were repressed by LPS [16]. BmToll9-1 interacted with BmSpz2 and activated the expression of AMPs [23]. Our recent publication indicates that BmPGRP-L4 negatively regulated the AMPs production [22]. BmPGRP-S5 functioned as a negative regulator of the AMPs pathway [33]. Starvation activated AMP genes expression via the insulin-like signaling pathway [34]. Serpin-4 negatively regulated prophenoloxidase activation and AMPs synthesis [35]. Scavenger receptor C regulated AMP expression by activating Toll signaling [36]. In addition, the overexpression of *DmToll1* and *DmToll9* in *D. melanogaster* significantly induced the expression of downstream AMPs [37,38]. 

## 5. Conclusions

Our study shows that BmToll9-1 might play a role as a positive regulator in the immune response of the Toll signaling pathway. BmToll9-1 might function mainly in the gut of the silkworm to maintain microbial homeostasis. Silencing of *BmToll9-1* might interfere with this balance and affect the growth of the larvae. Additionally, the reduction in *BmToll9-1* expression downregulates the signaling genes of the Toll pathway and most of the AMP production, leading to decreased antibacterial activity in the hemolymph.

## Figures and Tables

**Table 1:** Primers used in this study.

Gene	Accession Number	Primer Sequence (5′-3′)
Primer for qPCR		
BmToll9-1	PP496203	F: CGCAGACCGTTGAGTACATG
		R: CCAGACTGTCGTACCTTGGT
BmTIF4A	DQ443290	F: TTCGTACTGGCTCTTCTCGT
		R: CAAAGTTGATAGCAATTCCCT
BmTIF3s4	DQ443238	F: ACTTCAAGTTCAGGGCAGAT
		R: TTAATTGTTTTGTGGAGGCT
Signaling		
BmMyD88	XM_028186400	F: AACGGTCACGACTCGAACTC
		R: TCTGCCCAGATTCTTCATCC
BmTube	XM_028173146	F: GGCAGAAAGTTATGGCTTGG
		R: ATCCTCAAATGCTCGCTGTT
BmPelle	XM_028182154	F: ACATCAAGCCGGCTAACATC
		R: ACCGTGAGACCTTCAGATGC
BmCactus	XM_028180230	F: ACAGTCGTGCGTACATTTGG
		R: CAGCCTCTCCCTATCGTCAA
BmRel	XM_028175224	F: TCGAATACATCCCGGACTTC
		R: TGGAAGGTCCTTTCTTGCTC
BmTollip-v	XM_028186930	F: TGCTACTTCTGACGGTGTGG
		R: AGGGCCACTTTGTGGTACTG
BmPellino	XM_028184930	F: AGAGTCGCTCAGCACAACAA
		R: CAATGTGGCTCCACACAGAT
BmTRAF2	XM_028172769	F: TCGCTCCTATGGGCATAACT
		R: CCGCATGTTGTGATTACTGG
BmECSIT	XM_028171307	F: ATGCCGCCTTAGCTAGAATG
		R: GCCTTTGGGCAGTACGTCTA
Effector		
BmAttacin1 (BmAtt1)	NM_001043541	F: CAGTGAACTCGGATGGAACC R: GGCGCTGAGTACGTTCTTGT
BmCecropinA (BmCecA)	NM_001043997	F: CCGTCATAGGGCAAGCGAAA R: AGCAATGACTGTGGTATGTCAA
BmCecropinD (BmCecD)	NM_001043368	F: CTCCCGGCAACTTCTTCAA R: TTTGCCAGGGTGTCGACT
BmCecropinE (BmCecE)	XM_028187757	F: CGGAACCGAGATGGAAGATT R: TGGTCCAGCCTTGATTATCC
BmDefensin (BmDef)	AB_367525	F: GTTAAGTGCGGCGTTGACTG R: TGACAGGGAAAGTGGAAGGG
BmGloverin1 (BmGlv1)	AB_289654	F: GCTGGGATAGAAGCATCAGC R: ACATCAGGCCTTCTGTGACC
BmGloverin2 (BmGlv2)	NM_001044218	F: GGCTTACGGTACCAGGGTTT R: TGGCTTGTGCATTCTTGTTC
BmGloverin3 (BmGlv3)	NM_001099842	F: GGCCAACAAGAACGCACAAG R: ATCAAGATCCCACACACCGG
BmGloverin4 (BmGlv4)	AB_289657	F: GCAGATCTGGAATGACAGCA R: GTGACCGAATTCCTTCGAGA
BmMoricin (BmMor)	AB_006915	F: TGTGGCAATGTCTCTGGTGT R: CTGGCGATATTGATGGCTCT
BmMoricin-like (BmMorLB)	HQ204039	F: TCACTACATCTTCATACGCGAC R: TAGTTTATGTATTGGTTGTAGT
BmLebocin3 (BmLeb3)	NM_001126260	F: CTCGATCCAAACCGAAGGTA R: CGGCTGGTCAAGTCCAGTAT
BmEnbocin (BmEnb)	FJ373019	F: ACCTCGCACAACTAGTTCGG R: CCAACAGAACAAACCCACTCG
BmLysozyme (BmLys)	NM_001043983	F: TAACGGCTCGAAGGACTACG R: GAGGTCGGAGCACTTAACGT
Lysozyme-like protein (BmLLP3)	XM_012696687	F: GTTTAATCGAGCAGGGCAGC R: CACCCTTGCGACCTTCTTTG
BmPhenoloxidase inhibitor (BmPOI)	XR_001139981	F: GGATACGTGACTGGAAATGCA R: GTCATAATCCACGGGTTTGTCC
Prophenoloxidase 1 (BmPPO1)	AF_178462	F: AGTGGGAAGCCATTCTCCTT R: GCCAGGTTTCACTCCTTGAG
Prophenoloxidase 2 (BmPPO2)	XM_028180711	F: CCATTCTTCTACCGCTGGCA R: CGGGTTCTCGAGCTCAGATC
Nitric oxide synthase1 (BmNOS1)	XM_012689821	F: TCATCACCACTAGCGCATCC R: CCTTGTCCGTTCTGTGTCCT
Nitric oxide synthase2 (BmNOS2)	XM_012690766	F: ACAACAGACGCCACATCCAT R: AAATTCGGGTAAGCGCTCGA
Primer for dsRNA synthesis		
T7-BmToll9-1		F: TAATACGACTCACTATAGG ACTATAGGCACAGGTCGGGT
		R: TAATACGACTCACTATAGG TCGTTGTCCCATTCGCTGAT
T7-GFP		F: TAATACGACTCACTATAGG TACGGCGTGCAGTGCT
		R: TAATACGACTCACTATAGG TGATCGCGCTTCTCG

## Data Availability

The raw data supporting the conclusions of this article will be made available from the corresponding author upon reasonable request.
